# Medicine

**Published:** 1909-06

**Authors:** J. Michell Clarke


### Progress of the Medical Sciences.

### MEDICINE.

**Aphasia.**—The following is taken from a very excellent review of the recent work on aphasia by Dr. James Collier.[1]

Marie in 1906 strongly attacked the accepted teaching as to the pathological basis and nature of disturbances of speech, and said that the whole subject was in urgent need of revision. This started a controversial discussion, which is not yet closed, and has been of material service in clearing up obscurities. Prior to Marie's paper it had been proved that the localisation of the chief centre for speech is the L. temporal lobe, and this was generally accepted. It was also held that motor aphasia was caused by a lesion of Broca's convolution in the L. 3rd frontal gyrus (F. 3), the signs being loss or disturbance of spoken language, with some interference with internal speech, associated generally, but to a varying degree, with alexia, agraphia and intellectual defects.

[1] *Brain*, 1908, xxxi, 523.

Sensory aphasia (Wernicke) was held to arise from a lesion of the posterior two-thirds of L. second temporal gyrus (T. 2) (Wernicke's zone), and perhaps also of surrounding gyri, it was characterised by defects of internal speech and intelligence, more or less complete "word-deafness" and "word-blindness," and often by severe defects of outgoing speech, paraphasia and jargon-aphasia. The chief difficulty has been in the accurate differentiation of these two types of motor aphasia and sensory aphasia, for the difference between them is rather one of degree than of kind; they are often difficult to distinguish clinically, and have been repeatedly confused, the same cases being claimed by one author as "motor" and by another as "sensory" aphasia. About the types "pure motor aphasia" and "pure word blindness" there is no dispute, the former depends upon a subcortical lesion isolating the speech-centres upon the outgoing side, and the latter upon a similar lesion isolating the speech-centres on their incoming side from the primary visual centres in either hemisphere. The term "total aphasia" is applied to cases where lesions occur in both Broca's and Wernicke's regions, and is characterised by severe defects of intelligence and of all the elements of speech.

It was on account of the complication of the subject, the hypothetical nature of the foundations on which the various schemes of aphasia were based, the inaccuracy of much of the pathological work, and the absence of a working hypothesis universally and easily applicable for the study of clinical varieties that Marie, in 1906, called for consideration of the whole material.

Marie disputed the view so generally held that the building up of the faculty of speech depends upon the registration of speech memories in the central cortex as "visual word," "auditory word," and "kinæsthetic word" memories. He denied that Broca's convolution F. 3 has any function in connection with speech, and that there is any distinction between "motor" and "sensory" cortical aphasias. He says that there is only one speech-centre, diffusely localised in the L. temporo-parietal lobe, and that this centre is a region of intelligence specialised for language, not a centre of sensory images; "word-blindness" and "word-deafness" are thus defects of the special intelligence for speech. There is but one variety of aphasia from lesion of the cerebral cortex, and this corresponds with the aphasia of Wernicke, resulting from lesion of the region of intelligence specialised for language seated in Wernicke's zone. The classical aphasia of Broca is a combination of aphasia plus anarthria, resulting from a double lesion in Wernicke's zone, and in a quadrilateral area around the lenticular mucleus. This quadrilateral is limited anteriorly and posteriorly by vertical frontal planes passing through the anterior and posterior limiting sulci of the insula, without by the surface of the insula, and within by the lateral ventricle. It is connected with F. 3 in front, and by a compact band of fibres (temporo-parietal isthmus) with Wernicke's zone. Lesions in front of this isthmus produce anarthria, behind it aphasia. It is to be noted that Marie employs the term anarthria not according to the general use, as a paralytic defect of articulation, but in the sense of Broca's aphasia to mean "loss of, or difficulty with, articulate speech, with complete preservation of internal speech, and with the absence of articulatory troubles of a paralytic nature."

Naturally these views of Marie have aroused much controversy. With regard to his contention that Broca's convolution F. 3 has nothing to do with the speech mechanism, Dr. Collier quotes cases from Déjèrine (and others) in which very careful pathological and microscopical examination showed a lesion of this convolution in connection with the clinical features of Broca's aphasia, and also points out the important bearings which the recent work on apraxia,[1] dependent as it probably is on lesion of the first and second left frontal convolutions, has upon Broca's aphasia. He concludes, after careful weighing of the evidence and arguments on both sides, in favour of the retention of the L. third frontal gyrus as an important speech-centre upon the outgoing side. He points out the difficulties of the unlocalised and functionally unexplained motor mechanism in the "quadrilateral" area, and suggests that the "anarthria" ascribed to lesions of it may arise from section of the projection of F. 3 and of the foot of the anterior part of the corona radiata which are contained in this quadrilateral. It further seems unlikely that the lenticulo-striate body (quadrilateral), which has little or no direct connection with the cortex cerebri, should be a motor mechanism for speech, one of the highest cerebral functions, and Dr. Collier considers "that Marie has not proved his case for the shifting of the motor speech function from Broca's region to the quadrilateral, yet the question is one that needs much further investigation."

Dr. Collier thinks that the most important part of Marie's work is his conception of the speech function of the cerebral cortex. Marie does not believe that the elements of speech are registered as sensory images, and considers that the separation of the visual from the auditory part of speech is erroneous, and that this separate localisation in the cortex has no foundation in fact. The function of speech is diffusely localised in the L. temporo-parietal region, an intellectual centre specialised for speech, not a sensory centre foı the storing of sensory images. Destruction

[1] Apraxia is the inability to perform skilled movements with the limbs in absence of paralysis, of loss of sensation or of co-ordination, or of intellectual defect. Motor apraxia thus bears the same relation to movements of the limbs as motor aphasia to movements of the muscles concerned in speech.

of individual portions of this area causes " a general depression of speech intelligence and of general intelligence, which results in the loss of the speech functions in reverse order of their depth of impression " or acquirement. The whole of the phenomena of aphasia are much more easily explained on the ground of varying degrees of crippling of a single speech intelligence centre than upon the old hypotheses."

**Anaphylaxis.**—In an interesting paper Dr. Eugene McCampbell[1] discusses this subject. In a certain percentage of cases in which diphtheria antitoxin is injected toxic symptoms occur, and occasionally death. These untoward results might result either from the action of antitoxin itself, or from the horse serum in which it is contained. Experiment has proved that the antitoxin is not the poisonous body, and that there is no toxin or toxon present. Rabbits, guinea-pigs, and other animals can withstand very large injections of normal horse serum without ill-effect. If, however, a guinea-pig is given, say, .004 cc., and then a larger injection of, say, .1 cc. horse serum the animal dies or is seriously ill after an interval of 8–13 days. Death occurs, as in antitoxin poisoning in man, from paralysis of respiration. The guinea-pig has been rendered hypersensitive to horse serum, so that a second somewhat larger injection produces serious or fatal symptoms. The second injection must be made at the proper interval, 8–13 days; if given before this the reaction does not occur, and on being injected subsequently at the proper interval again no reaction occurs (immunity). To this condition of hypersusceptibility or hypersensitiveness to proteins the name anaphylaxis is given. Small injections usually hypersensitise, large produce immunity. Two well-known examples of anaphylaxis are the " reaction " to tuberculin and mallein by patients the subjects of turberculosis and glanders respectively. Very many if not all proteins (including various species of bacteria, yeasts), except gelatin, will produce the reaction. Further, a guinea-pig may be sensitised to several proteins at the same time. If the blood serum of a sensitised animal be injected into a second animal the latter is rendered " anaphylactic " more speedily than the first. The sensitising substance is in the serum, and it is not necessarily present in large amounts. Neither the toxic nor the sensitising substance have been isolated. Wells has shown that one millionth of a gram of pure egg-white (triple recrystallised) will sensitise a guinea-pig fatally. The very small amounts of egg-white which will sensitise and intoxicate proves that it is the same molecule or two molecules of the same kind. Probably, therefore, in the case of egg-white and of bacterial proteins the same element sensitises and poisons. In the case of horse serum there is no such positive

[1] "Anaphylaxis and Immunity," *Med. Rec.*, 1909, lxxv, 555.

proof; probably the sensitising substance is euglobulin or a closely allied body.

The anaphylactic reaction is a specific one to the protein to which the animal is sensitised. Instead of injections, guinea-pigs may be sensitised by feeding them either with serum or *uncooked* meat; thus fed on dried horse meat they were sensitised to horse serum.

Anaphylaxis can be transmitted through the mother to the offspring; in most cases it is a case of antenatal acquirement and not true heredity. The transmission of anaphylaxis to bacterial proteins from the mother to the young is a possible explanation of inherited tendencies to definite infectious diseases, *e.g.* tuberculosis.

An individual may become immune by means of a process of anaphylaxis. The first injection of a bacterial protein into an animal produces no effect, but the second injection, after an interval of time, produces the same anaphylactic symptoms as horse-serum. If the animal recovers it is usually immune to further injections with this protein. By this phenomenon the period of incubation in some infectious diseases which last from ten to fourteen days is explained. In those having a shorter period, such as pneumonia, the crisis corresponds to the period of sensitisation.

In pregnancy the similarity of the symptoms and the conditions under which eclampsia occurs suggest anaphylaxis as an explanation. The possibility of the mother being sensitised by either the proteins of the placenta or the fœtal blood and subsequently being poisoned thereby has been suggested. Experimental evidence seems so far to show that in guinea-pigs the mother may be sensitised by the autolytic products of her own placenta.

**Sensitisation of bacteria.**—A research by Drs. White and Graham[1] on the interaction of micro-organisms, normal serum and leucocytes, and its effect on infection is of interest. They conclude from their experiments:—

(1) That the infective power of tubercle bacilli for rabbits is greatly increased by sensitisation with normal rabbit serum.

(2) This sensitisation may be accomplished by minute quantities of serum (.1 cc.), and greatly increased by previous incubation of the serum and bacteria at 37° C. in vitro.

(3) The increased infective power may be counteracted by exposure to leucocytes of the same species.

(4) The power of sensitising bacteria, and thereby increasing

[1] "On the increased infective power produced in bacteria by sensitisation with normal serum of the same species," *J. Med. Research*, 1909, xx, 67.

their infective power, is probably possessed alike by homologous and heterologous normal and immune serum.

(5) That probably other bacteria are also capable of sensitisation, and their infective power thereby increased.

J. MICHELL CLARKE.

### SURGERY.

